# Supplementary material for: Verification of Outer Hair Cell Motor Protein, Prestin, as a Serological Biomarker for Mouse Cochlear Damage
Source: Int J Mol Sci. 2024 Jul 2;25(13):7285. doi: 10.3390/ijms25137285 (PMC11241755; doi:10.3390/ijms25137285)
Supplement: Supplementary file 1 [file ijms-25-07285-s001.zip › ijms-3029425-supplementary.pdf]

Supplementary Materials for

Verification of Outer Hair Cell Motor Protein, Prestin, as a Serological Biomarker for Mouse Cochlear Damage

Jing Zheng, Yingjie Zhou, Robert J. Fuentes and Xiaodong Tan

The PDF file includes Figures S1 to S2.

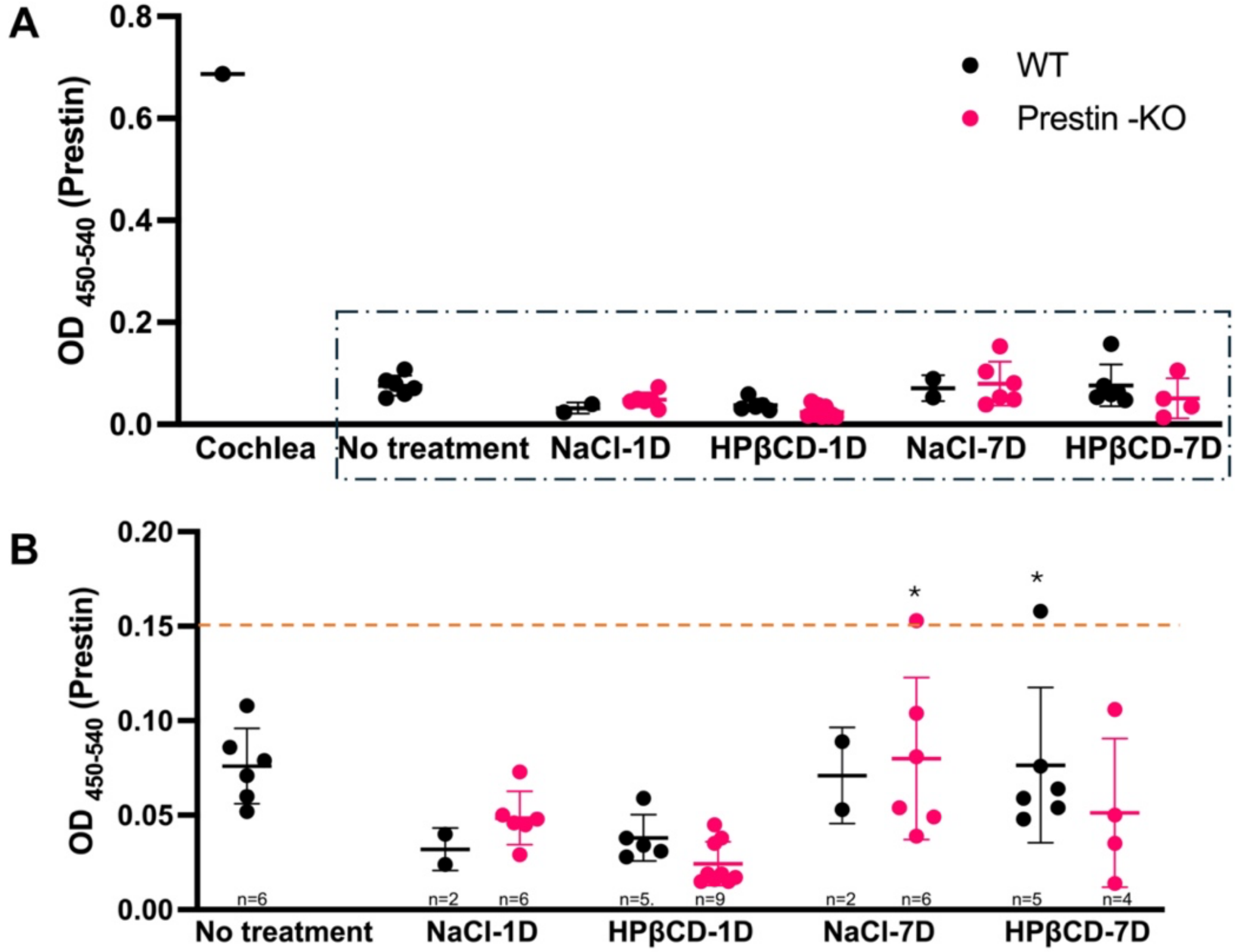

Figure S1. Prestin levels were measured using an ELISA kit from MBS. **A**. Prestin was detected in a cochlear homogenate (a positive control) but undetectable in bloodstream samples. **B** is an enlarged portion shown in the boxed region in **A**. Plasma or serum samples were collected one day (1D) or seven days (7D) after HPβCD or NaCl injection. The dashed line shows the limitation of detectable Prestin concentration by ELISA (15.6 pg/ml). Prestin concentrations in the bloodstream from both WT and *prestin*-KO mice were below the detectable limit of Prestin-ELISA regardless of whether mice were treated with HPβCD or NaCl. Each dot represents one serum sample, which might be derived from the same animal with different degrees of hemolysis. Two samples with hemolysis ($OD_{414} > 0.5$) were labeled with '*'. The numbers of samples (n) and means ± SD are shown.

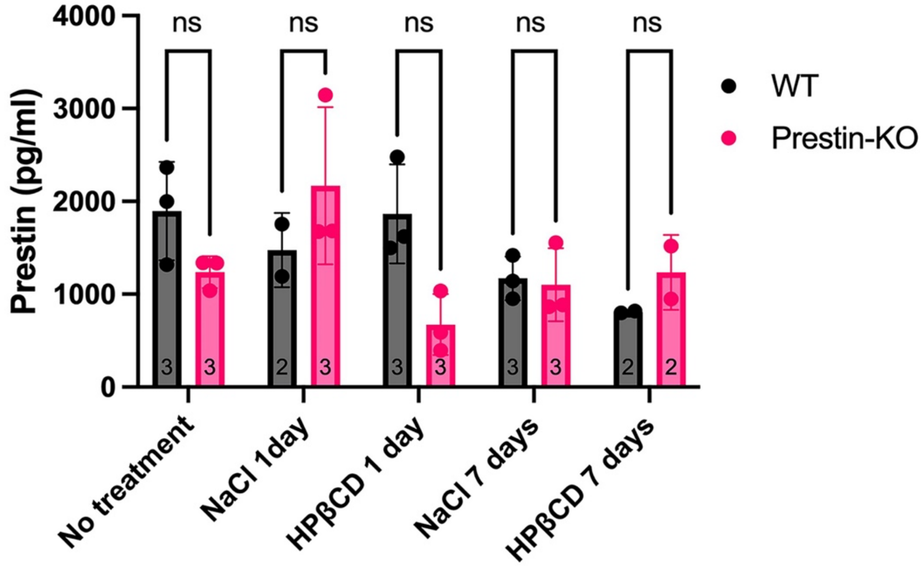

Figure S2. Prestin levels were measured using a more sensitive ELISA kit from MBS. WT and prestin-KO mice were injected with either HPβCD, NaCl, or no injection at all (no treatment). Serum samples were collected one day or seven days later for the MBS kit with a detectable range of 7.8-500 pg/ml. The number of samples were shown. Each dot represents one animal sample. Means ± SD were plotted, and significance was determined using Mixed-effects model (REML), and unpaired t-tests for the MBS ELISA. ns: not significant.

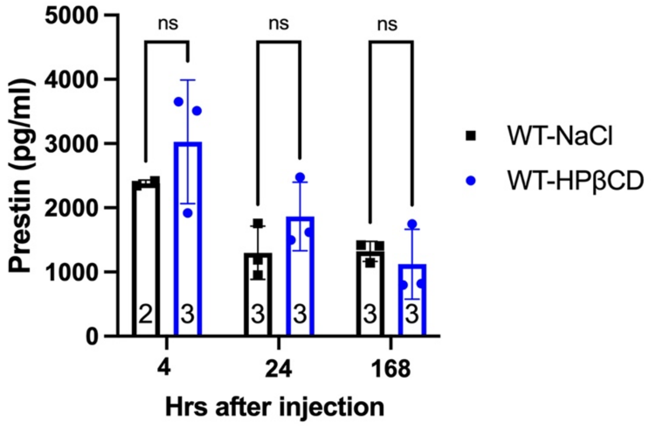

Figure S3. Prestin levels in serum samples of WT injected with HPβCD or 0.9%NaCl. The serums were collected at different time points after littermate WT mice were injected with HPβCD or 0.9%NaCl. Prestin concentrations were measured using a mouse Prestin-ELISA kit made by MBS. Each dot represents one animal sample. The number of samples and *p* were also shown. Means ± SD were plotted, and significance was determined using multiple unpaired *t*-tests. ns: not significant.
